# Synthesis and application of Cobalt-Silver nanohybrid for antimicrobial wastewater treatment and agricultural productivity enhancement

**DOI:** 10.1038/s41598-025-99333-w

**Published:** 2025-05-10

**Authors:** Sayed M. S. Abo El-Souad, Marwa A. Ramadan, D. Zahran

**Affiliations:** 1 Department of Botany and Microbiology, Faculty of Science, Cairo University, Giza, 12613 Egypt; 2 Department of laser application in metrology, photochemistry and agriculture, National Institute of Laser Enhanced Science (NILES) Cairo University, Giza, 12613 Egypt

**Keywords:** Metallic nanocomposite, Antibacterial, Antifungal, Water treatment and wheat growth enhancement, Microbiology, Environmental sciences, Hydrology, Nanoscience and technology

## Abstract

1- This work emphasises the potential of Co@Ag-NPs as an efficient antimicrobial agent. The scientific community has recently shown silver nanohybrids to maintain plural consistency and their potential applications in wastewater treatment. Where these nanohybrids showed highly removing capacity of the three main contaminants (pesticides, microorganisms, and heavy metals) from waste water. The ability of silver and cobalt nanohybrids to inhibit bacteria and fungi that cause illnesses both in vitro and in vivo has made them an outstanding antimicrobial agent. Cobalt-silver nanohybrid particles (Co@AgNPs) have antibacterial properties against both Gram-positive and Gram-negative bacteria, including those that are resistant to multiple drugs. Co@AgNPs have several simultaneous modes of action, and when combined with organic chemicals or medicines that fight bacteria, they have demonstrated a synergistic effect on infections. Because of their unique properties, silver and cobalt nanohybrids can be used in medical and healthcare goods to effectively treat or prevent infections. The preparation and characterization of highly stable cobalt silver nanohybrid (Co@Ag) have been reported. Out of the water samples, four bacterial and seven fungal isolates are identified. Various concentrations of Co@Ag, ranging from 10^− 1^ to 10^− 3^, have been seen to impact and produce varying diameters of inhibition zones in bacterial isolates Shigella, Salmonella, E. coli, Pseudomonas aeruginosa and fungal isolates Aspergillus flavus var columnaris, and Aspergillus awamori. Water samples treated with Co@Ag nanoparticles when plated on LB and Czapek Dox agar did not show any growth of bacteria and fungi after five and seven days of incubation, respectively. Furthermore, data demonstrated that shoot and root length and germination percentage of wheat seeds irrigated by treated water increased progressively from 7.5 cm to 9.2 cm, from 9 cm to 11 cm and from 90 to 100%, respectively, as Co@Ag concentrations were elevated from 0 to 10 and 20 mg/l.

## Introduction

Water is crucial for life, covering 70% of the planet’s surface. However, rapid population growth, industrialization, urbanization, and agricultural practices are causing degradation of water supplies. Approximately 1.2 billion people lack access to clean drinking water, 2.6 billion struggle with basic sanitation, and millions die from diseases caused by contaminated water. Treatment techniques for wastewater include physical-chemical, conventional, and biological processes^1,2^. These physicochemical methods of treating wastewater frequently require both energy and intensive mechanical methods (such as ozone, permanganate, ion exchange resins and regenerates, coagulation and filtration aids, ammonia, alum, sodium hydroxide, ferric salts and hydrochloric acid) and chemically intensive mechanical methods, which calls for infrastructure and engineering expertise. Furthermore, it has been noted that conventional methods are insufficiently effective in fully eliminating heavy metals, phosphorus, nitrogen, and toxins from contaminated wastewater. All of these elements have their own benefits and drawbacks, and even though they made them costly and time-consuming, they all reduce the levels of various pollutants^2,3^. Fundamentally, nanotechnology involves the matter manipulation at the molecular and atomic levels in order to create novel systems, devices, and structures with enhanced mechanical, electrical, optical, conductive and magnetic qualities^4^. Nanotechnology is being investigated as a potentially useful technology and has shown impressive results in different areas, including treatment of wastewater^5^. Nanoparticles such as magnetic nanoparticles, carbon-based nanoparticles, organic polymer nanomaterials, transition metal sulfide nanoparticles, metal nanoparticles, metal oxide nanoparticles, silica-based nanomaterials and biogenic nanoparticles have been used for the remediation of pollutants such as microbes, antibiotics, other pharmaceutical compounds, heavy metals and organic compounds^6–9^, depending on the affinity of each nanoparticle for the contaminants. Because of their small size, high surface area, and simplicity of functionalization, nanostructures have unmatched possibilities for creating more potent catalysts and redox-active media for purification of wastewater. Various contaminants, including biological toxins, organic and inorganic solvents, color, heavy metals and microorganisms that cause cholera and typhoid, have been reported to be effectively removed from wastewater by nanomaterial^10,11^.

Silver nanoparticles (AgNPs) have highly reactive surfaces indicate that they may have a future in applications of antimicrobial^12,13^. AgNPs have the ability to kill microorganisms through a variety of potential antimicrobial mechanisms, including electron transport, DNA damage, Ag + ion release, and rupture of cell membranes. AgNPs are highly regarded for their oligodynamic performance, minimal environmental toxicity, and broad-spectrum antimicrobial capabilities. AgNP-based goods include antimicrobial coatings for devices and surgical instruments, diabetic foot and wound dressings, antibacterial soaps, and creams. It is also used in portable clean water filters^5,14^. AgNPs are nearly harmless at low concentrations, but when they build up in mammalian cells, they can interact with the body to induce infections and adverse effects such argyrosis and argyria. As a result, eliminating silver nanoparticles away from the body is a difficult but necessary task^15,16^. Additionally, the penetration of possible cytotoxic effects in a human cell can be increased by using smaller nanoparticles (NPs), which are thought to be more effective bactericidal agents^17–19^. Different new approaches are needed to deal with this difficult situation, and one of them might be loading AgNPs onto magnetic cores to create nanocomposites^15,20–22^. Cobalt (Co), Iron (Fe), and Nickel (Ni) are the most well-known and alluring ferro-magnetic elements that are part of the third-block of magnetic materials. They are frequently employed in the creation of nanocomposite structures as magnetic cores because of their superior magnetic properties in their elemental forms. The saturation magnetization (Ms) values for Fe, Co, and Ni at room temperature are 220, 170, and 55 emu g − 1, respectively, whereas the Curie point (TC) values are 770 °C, 1131 °C, and 358 °C, respectively. Fe has a greater Ms value than Ni and Co, which makes it more magnetized, but its larger negative reduction potential (E0) value indicates that it is unstable because of its rapid oxidation in air. Fe nanoparticles are more susceptible to oxidation than the other two elements because of their higher surface to volume ratio, which intensifies the oxidation process at the nanoscale. With respect to Ni, Co is a better choice for the core material of hybrid nanostructures due to its slightly larger magnetization, higher Curie point, and greater magnetocrystallinity. The core material has been used in a variety of nanocomposite materials is (Co), such as cobalt–silver (Co@Ag), cobalt–copper (Co@Cu), cobalt–gold (Co@Au), and cobalt–platinum (Co@Pt) nanoparticles. Two notable benefits of using Co as a core material with Ag, Au, and Cu (non-ferromagnetic materials) are high stability at higher temperatures and giant magnetoresistance (GMR), which in turn increase their antimicrobial activities under a wide range of conditions. These properties are desirable in media recording, multifunctional sensing and wideband photovoltaic solar cell applications. Bimetallic hetero-nanostructures can be created using a variety of chemical, physical, and biological techniques; however, the solution phase chemical reduction strategy can be useful because of its straightforward handling and economy of cost. Additionally, it offers excellent thermal stability, purity index, and control over customizing the generated nanostructures attributes by adjusting particle size without the need for complex equipment^23–26^. Silver nanoparticles have been shown to have beneficial effects in a number of economic areas, including agriculture. Over the past few years, the scientific interest in AgNPs in plant biotechnology and agriculture has demonstrated their maximum effectiveness. In addition to improving plant growth and seed germination, it also raises the photosynthetic process’s quantum efficiency. AgNPs are essential to agriculture because of their many uses, which are critical for increasing crop yield and guaranteeing food security. Additionally, they function as nano-pesticides, giving the target plants an adequate dosage without needlessly releasing pesticides into the environment. In order to prevent excessive nutrient loss, nano-fertilizers release nutrients to the plants gradually. AgNPs are a great tool for safely controlling pests because they are used for non-toxic and efficient pest management. AgNPs, a blend of edible and non-biodegradable polymers, offer various biological properties for active food packaging and plant growth protection, with potential agricultural applications^27^.

Our study aims to use cobalt–silver (Co@Ag) nanohybrids to treat waste water from bacterial and fungal pathogens, after that use these water in germination of Wheat seeds as a clean water source.

## **Material and method**

### Sampling

Two distinct drain resources outlet points have been chosen to conduct the investigation. Standard procedures were used to analyze agricultural drainage water from the GIZA Governorate, we used stagnant wastewater that cannot be used for drinking or irrigation and highly contaminated with microbial infection.

The locations of samples were 30°01’38.5"N 31°12’25.1"E and 30°01’39.4"N 31°12’27.4"E.

#### Isolation and purification of bacterial and fungal isolates

Bacteria and fungal isolates were isolated from each water samples and plated on Luria Agar (LA) and Czapek Dox Agar from the total number of viable bacterial and fungal count plates. Each plate’s growing colonies were distinguished and grouped based on cultural traits before being purified and kept for additional identification. The isolated bacteria were identified using phenotypic techniques (colony morphological studies, Gram-staining, and VITEK 2 system) for identification, where fungal isolates identified by using morphological studies^28^. In order to isolate bacteria and fungi, we first inoculate particular medium with 1 ml of wastewater samples and incubate for 24 h for bacteria and 6–7 days for fungi. Once isolates are purified, we test the antimicrobial activity of this nanoparticle.

## Co@Ag Preparation and characterization

As mention of our previous works^15,22^ Co@Ag prepared in two steps. Firstly cobalt nanoparticles (CoNPs) prepared by reduction method of metal salt, where 1 gm poly vinyl alcohol (PVA) dissolved in warm water (20 ml) after complete dissolve, 3 ml of 0.05 M Co were added with continuous stirring for 15 min. Then 5 ml (0.05 M) sodium borohydride drop wised with stirring until become completely dark. The second step is coating CoNPs with AgNPs by reducing silver nitrate in the presence of CoNPs which act as nucleation sites for the resultant Co@Ag. In this step 7 ml of the prepared CoNPs were stirred with 7 ml (0.05 M) AgNO_3_ for 15 min in dark conditions, then 5 ml (0.05 M) sodium borohydride was added drop wise under the stirring. A dark yellowish color results from the reduction process and denotes the creation of Co@Ag. For characterization of Co@Ag different techniques were used. UV-Vis-NIR spectrophotometer (Cary 5000, Agilent, Santa Clara, USA) in the wavelength range of 200–900 nm was used to examine the characteristic absorption peak of Co@Ag. JEM-1400 TEM used to examine the morphology of the sample. Vibrating sample magnetometer (VSM) EV11 (Model 8810), ADE Technologies, Inc. was used to examine the magnetic properties of Co@Ag.

## Antimicrobial activity of Co@Ag

The Kirby-Bauer disc diffusion susceptibility test method^29^ was used to measure the antimicrobial activity of Co@Ag against the isolated bacterial and fungal strains. Using sterile cotton swabs, the bacterial strains (0.5 McFarland) were inoculated onto Mueller-Hinton agar (MHA) (Merck, Germany). After that the discs were loaded with 10 µL of synthesized Co@Ag individually, and the zones of inhibition were detected after 18 h at 37 °C, where the fungal strains spread across the Cpazek Dox Agar and 10 µL of chemically produced Co@Ag loaded on sterilized discs, after 4 days at 28 °C an inhibitory zones were measured. Vancomycin (20 µg/ml) and miconazole (10 µg/ml) used as positive control and distilled water used as negative control.

## MIC technique using resazurin

### Preparation of bacterial suspension

Stock cultures of *Salmonella*,* E. Coli*,* Shigella*, and *Pseudomonas aeruginosa* were subcultured onto BA plates and incubated at 37 °C for the whole night. The next day, three to four different but similar-looking bacterial colonies were added to 10 millilitres of sterile Mueller Hinton broth (MHB) for inoculation. After that, the inoculation was kept at 37 °C overnight. The overnight bacterial suspensions were adjusted to 0.5 McFarland Standard using sterile MHB broth. In order to make comparisons easier, the bacterial suspensions were corrected to the density of the 0.5 McFarland Standard using black lines that contrasted with white.

### Resazurin solution Preparation

The resazurin solution was made by dissolving a 270 mg tablet in 40 millilitres of sterile distilled water. A vortex mixer was used to ensure that the fluid was dissolved evenly.

## Turbidometric assay based on résazurin and determination of minimum inhibitory concentration (MIC)

Using the resazurin-based turbidometric (TB) assay to demonstrate the inhibitory effects on *Salmonella*,* Shigella*, *Pseudomonas aeruginosa*, and *E. coli*^30^.

## **MIC plates preparation**

Antibacterial assay based on microtitre plates that uses resazurin as a cell growth indicator and is used to screen nanoparticles for bacteria in vitro.

An aseptic atmosphere was used to create the plates. A sterile label was placed on a 96-well plate (Fig. 5). One hundred microliters of the test material were pipetted into the plate’s first row. For every well that remained, 50 µl of either ordinary saline or nutritional broth was added. Utilising a multichannel pipette, the serial dilutions were performed. After usage, tips were discarded so that each well contained 50 µl of the test item in progressively lower quantities. To every well, 10 µl of the resazurin indicator solution was added. Finally, 10 µl is needed to achieve a concentration of 5 × 10^5^ cfu/ml. Total well concentration (ranging from 100 to 0.0975%), as indicated in **(Table **3**).** Each plate has cling film loosely wrapped over it to keep microorganisms from drying out. Each plate had a set of controls, which included a column with all the solutions removed save the test chemical, another column with all the solutions removed but the bacterial solution, and a positive control of 10 µl of nutritional broth (usually vancomycin in serial dilution).

## Co@Ag and wastewater treatment

To evaluate the antimicrobial activity of nanoparticles, 18 ml of each wastewater sample and 2 ml of nanoparticle were added to a 100 ml conical flask. The mixture was then incubated at 30° C and 120 rpm for 2 days in a dark condition. One milliliter of the mixture was placed into different selective and differential media (LB, blood agar and mannitol salt agar) for bacteria, while the fungi were cultured in (Czapex Dox and Sabouraud agar) media.

### Application of treated waste water

The Co@Ag nanohybrids were mixed with waste water samples and incubated for 24 h at 30 °C and 120 rpm in the dark. After incubation nanoparticles were removed from treated water samples by centrifugation at 4000 rpm for 20 min. Wheat seeds (*Triticum aestivum L*.) (Gemmiza 12) were acquired from the National Agriculture Research Centre in Giza. They were then surface sterilized using a 10% chlorox solution and passed through three rounds of autoclaved distilled water washings. The seeds were placed on the filter paper in the petri plates. After moistening the filter sheets with a 5 ml of treated waste water, they were incubated for five days at 25–27° C in the dark. Following the germination of the seeds, the growth chamber was adjusted to a 16/8 h photoperiod, 24/20 2 ± 2 °C temperature, and 75/80% relative humidity. Treatments included: tap water (positive control); 20 mg/L Co@Ag suspension in waste water; 10 mg/L Co@Ag suspension in waste water; only waste water (negative control).

### Statistics

The experiments conducted in this work were replicated 3 times and the results obtained were analyzed using ANOVA one way, to analyze the statistical significance of differences between experimental values and control values, whereby the data was presented as a standard deviation (SD).

## Results and discussion

### Isolation

**Fig. 1:**
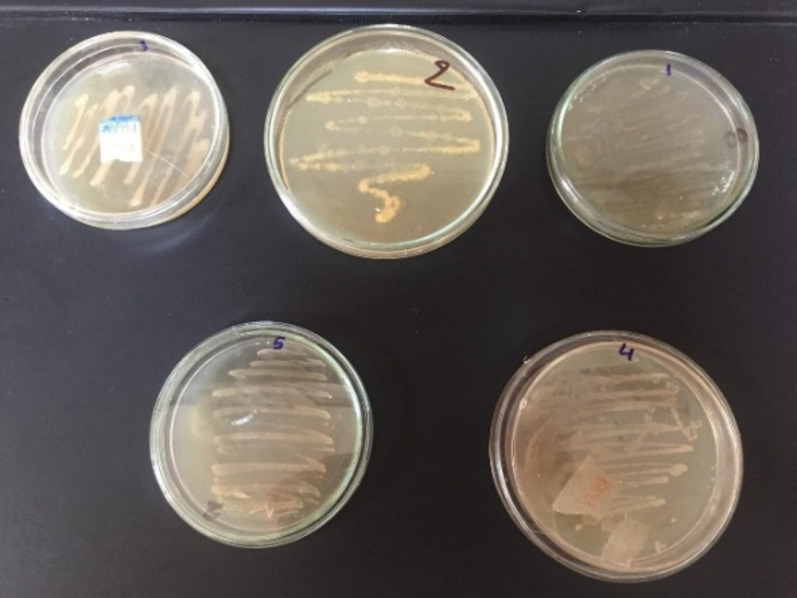
Different bacterial isolated from two waste water samples on differential media.

**Fig. 2:**
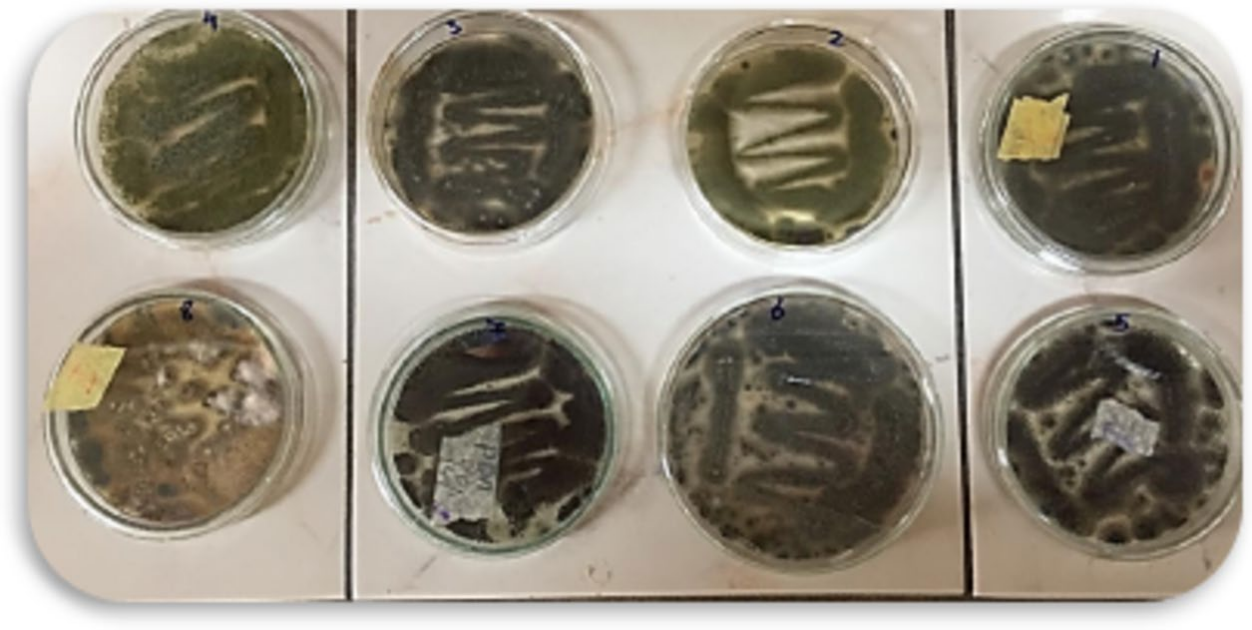
Morphological features of isolated fungi on Czapex Dox Agar.

In our study, four bacterial (Fig. 1) and seven fungal isolates (Fig. 2) were isolated from two different wastewater samples. These isolates were purified and maintained on LB and Czapex Dox agar for further investigation.

### Identification of bacterial isolates

Gram stain performed for the four different bacterial isolates and the results showed that all isolates are Gram –ve bacilli via direct inoculation of culture fluids from positive blood cultures into VITEK 2 ID-GNB cards, the Gram-negative bacteria detected by the conventional method were also examined. The investigation showed that the majority of bacteria were correctly identified when compared to the traditional method. As demonstrated by Table 1.

**Table 1:** Using VITEK^®^ 2 ID-GNB cards loaded with culture fluids from positive blood culture bottles, identify Gram-negative bacilli.

Bacteria	Isolate No.	Correct identified		VNGNB		Not identified		Misidentified	
		No.	Percent	No.	Percent	No.	Percent	No.	Percent
*E.coli*	**1**	**1**	**100**	**0**	**0**	**0**	**0**	**0**	**0**
*Salmonella*	**2**	**2**	**100**	**0**	**0**	**0**	**0**	**0**	**0**
*Shigella*	**3**	**3**	**98**	**0**	**0**	**0**	**0**	**1**	**2.7**
*Pseudomonas aeruginosa*	**4**	**4**	**100**	**0**	**0**	**0**	**0**	**0**	**0**

### Microscopic features isolated Fungi

The isolated fungi were investigated in this study based on their morphological, microscopic, and cultural traits. In this study, seven different fungal species were isolated and identified, and 1 isolate not identified, as shown in **Figure (**2**).**

Morphological and microscopic identification showed that the isolated fungal samples are *Aspergillus fumigatus* **fresenius 1863**, *Aspergillus flavus var columnaris* **Raper & Fennell 1965**, *Aspergillus aculeatus* **Iizuka 1953**, *Aspergillus flavus* **Link 1809 var flavus**, *Aspergillus awamori* **Nakazawa 1907**, *Cladosporium cladosporioides* (**fresenius) de vries 1952** and *Aspergillus carbonarius* (Bainier) **Thom 1916**.

### Co@Ag Preparation and characterization

**Fig. 3:**
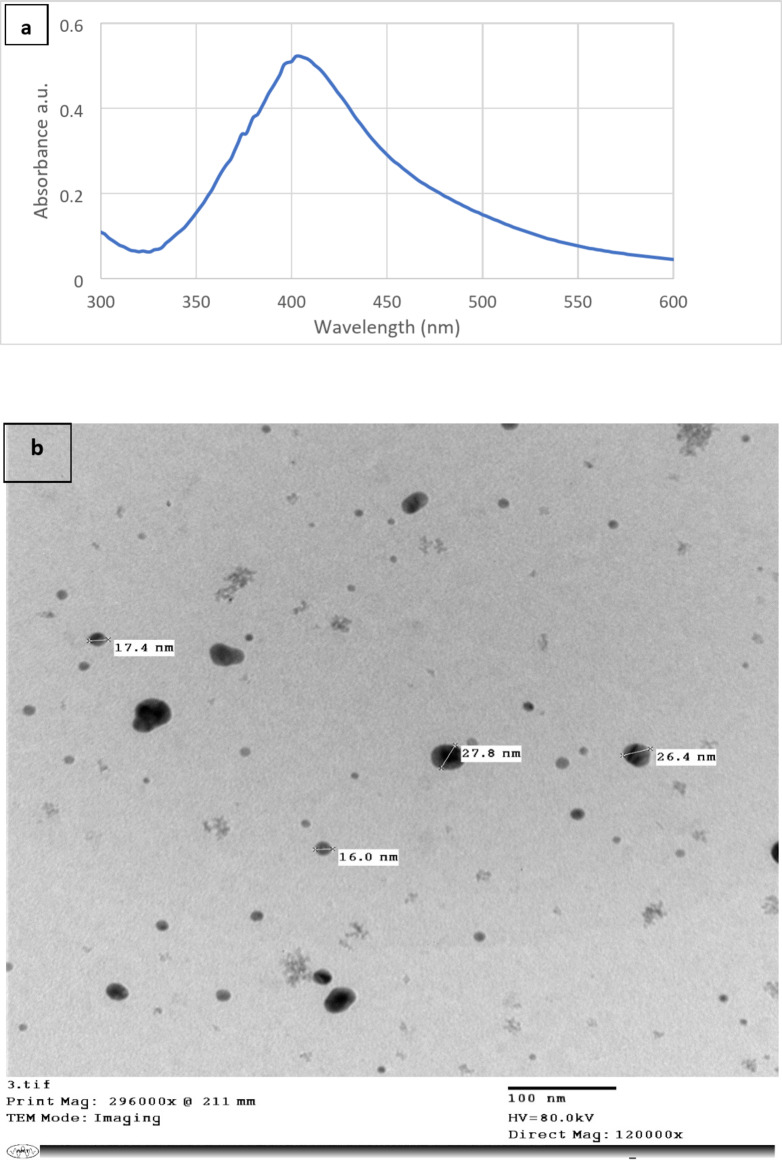

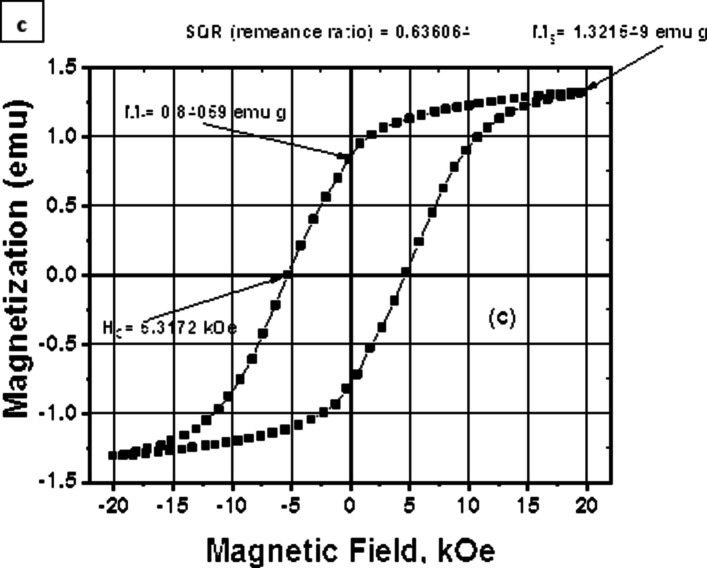
Co@Ag (**a**) Absorption spectra (**b**) TEM Image (magnification 100 nm).

As was indicated in the section of preparation, hydrogen gas generated from the hydrolysis of sodium borohydride reduces silver ions. In turn, this leads to the creation of Ag atoms, which diffuse to Co metals to form Co@Ag^5,14^. For several months, this nanohyprid remains remarkably stable in an aqueous solution without exhibiting any sign of agitation. As seen in Fig. 3a, the production of Co@Ag is facilitated by an absorption peak that has a sharp at around 410 nm. According to Fig. 3b’s TEM picture, Co@Ag is roughly 20 ± 5 nm in size. Utilizing a vibrating sample magnetometer (VSM), magnetic property measurements on Co@Ag were obtained. A hysteresis loop was created for every measurement, and from this loop, calculations were made for the saturation magnetization (Ms), remanent magnetization (Mr) and the intrinsic coercivity (Hc). As seen in Fig. 3c, the magnetic hysteresis loops for Co@Ag were measured at room temperature (300 K) using VSM. Co@Ag showed a remanence MR of 0.84059 emu/g, switching field (HC) of 5.3172 T (5317.2 Oe) and a broader hysteresis loop with a greater magnetic moment (MS) of approximately 1.321549 emu/g. About 0.636064 was the remanence ratio (MR/MS) or Squarence ratio (SQR).

### c) hysteresis loop

#### Antimicrobial activity of Co@Ag

Co@Ag was tested for their antimicrobial activity against four different species of bacterial pathogens that are Gram-negative: *Shigella*, *Salmonella*, *E. coli*, and *Pseudomonas aureus* and for fungi *Aspergillus fumigatus*, *Aspergillus flavus var columnaris*, *Aspergillus aculeatu*,* Aspergillus flavus*,* Aspergillus awamori*, *Cladosporium cladosporioides* and *Aspergillus carbonarius*.

**Fig. 4:**
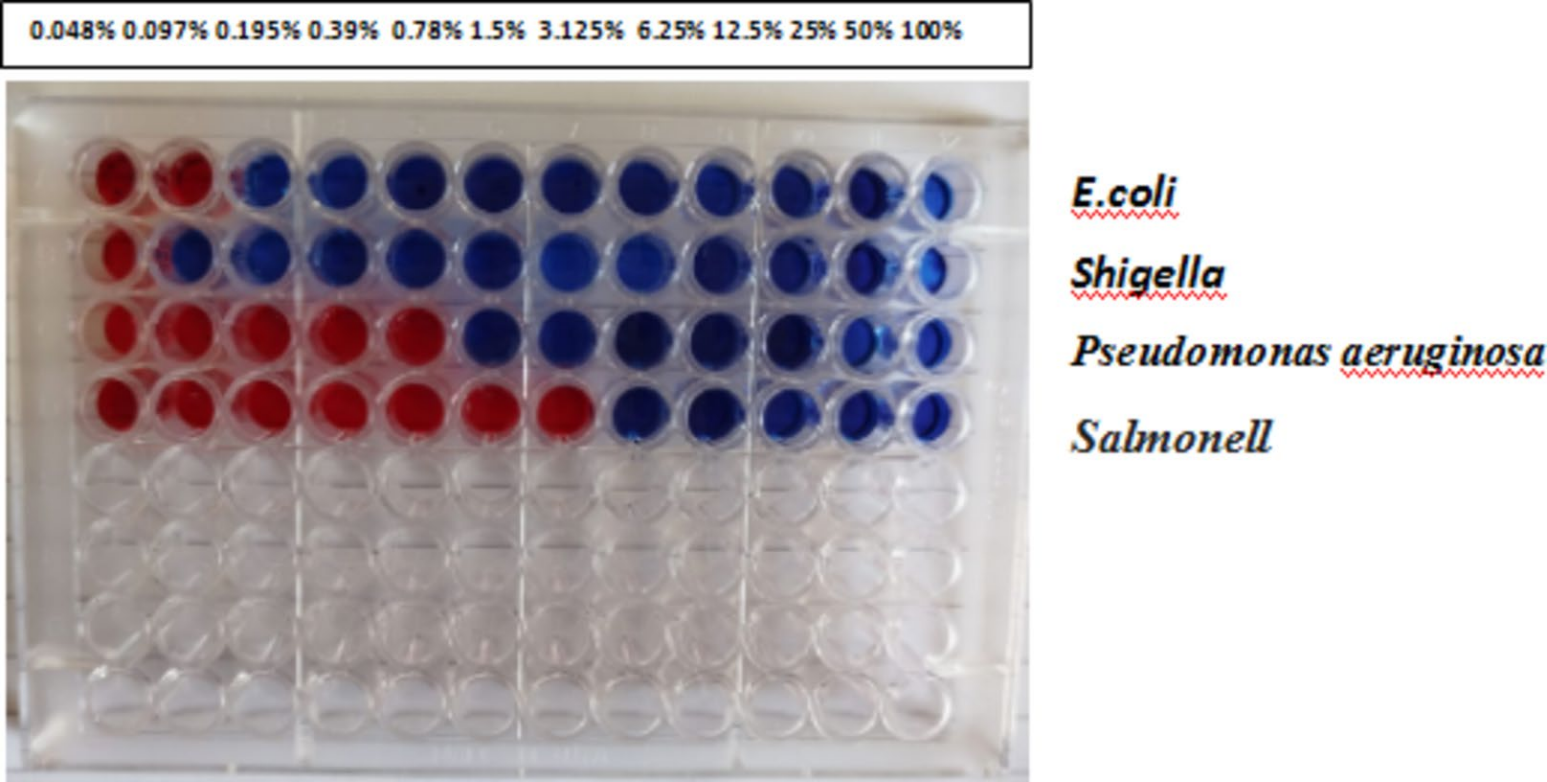
Microtitre plates using resazurin indicator to measure the antimicrobial sensitivity of the compounds utilized;.resazurin (blue) is the oxidized form and resorufin (pink) is the reduced form of resazurin).

Table 2 summarizes the Co@Ag disc diffusion test data. In the disc diffusion test, a clear zone surrounding the Co@Ag with different concentrations (10^−1^:10^−3^) disc indicated that the Co@Ag had antibacterial action, which means they might stop the growth of bacterial pathogens that are Gram-negative and pathogenic fungi. According to a prior work by^31^, Co@Ag have antimicrobial efficacy against Gram-negative bacteria and fungi. Figure 4 illustrates resazurin indicator to measure the antimicrobial sensitivity of the nanoparticles utilized; pink wells indicates reduced resorufin form, while blue wells indicates oxidized resazurin.

**Table 2:** The Co@Ag disc diffusion test results with their statistics value.

“Organism”	“Dilution”	“Diameter of clear zone (mm)”
*Shigella*	**10** ^**−1**^ **10** ^**−2**^ **10** ^**−3**^ **Vancomycin**	15.5^c^ ± 0.5 13.5^b^ ± 0.5 11.5^a^ ± 0.5 12.0^a^ ± 0.5
*Salmonella*	**10** ^**−1**^ **10** ^**−2**^ **10** ^**−3**^ **Vancomycin**	27^d^ ± 0.5 24^c^ ± 0.5 19^b^ ± 0.5 15.0^a^ ± 0.5
*E. coli*	**10** ^**−1**^ **10** ^**−2**^ **10** ^**−3**^ **Vancomycin**	6.5 ^b^ ± 0.5 6.0^ab^ ± 0.0 5.5^a^ ± 0.5 10.0^c^ ± 0.5
*Pseudomonas aureus*	**10** ^**−1**^ **10** ^**−2**^ **10** ^**−3**^ **Vancomycin**	22^c^ ± 0.5 19^b^ ± 0.5 12^a^ ± 0.5 13.0^a^ ± 0.5
*Aspergillus flavus var columnaris*	**10** ^**−1**^ **10** ^**−2**^ **10** ^**−3**^ **miconazole**	12^c^ ± 0.5 9^b^ ± 0.5 6^a^ ± 0.5 9.5^b^ ± 0.5
*Aspergillus awamori*	**10** ^**−1**^ **10** ^**−2**^ **10** ^**−3**^ **miconazole**	25^d^ ± 0.5 13^c^ ± 0.5 6^a^ ± 0.5 10.5^b^ ± 0.5

The mean is for triplicate measurements from one independent experiment ± SD, a-c means with different superscripts in the same column are considered statistically different (Duncan’s multiple range test, “p˂0.0001”)^32^.

### Minimum inhibitory concentration

Prepared chemical compounds were evaluated for their antimicrobial activity using MIC technique with resazurin, antimicrobial activity increased by increasing concentration of Co@Ag nanoparticles against bacterial isolates, *Salmonella* and *Pseudomonas aeruginosa* showed the highest MIC than *Shigella* and *E.coli* as showen in figure (4) and Table 3.

**Table 3:** MIC concetration for Co-Ag with antimicriobial activity on bacterial samples.

CHEMICAL	MIC	BACTERIA
Co-Ag	0.195%	*E.coli*
	0.097%	*Shigella*
	1.5%	*Pseudomonas aeruginosa*
	6.25%	*Salmonella*

### Co@Ag and wastewater treatment

By using different types of selective and differential media for demonstrate and count number of isolated bacterial and fungal isolates from wastewater treated with **Co@Ag** showing that no growth for bacteria after 5 days and fungi after 8 days, as showed in **figure (**5**).**

**Fig. 5:**
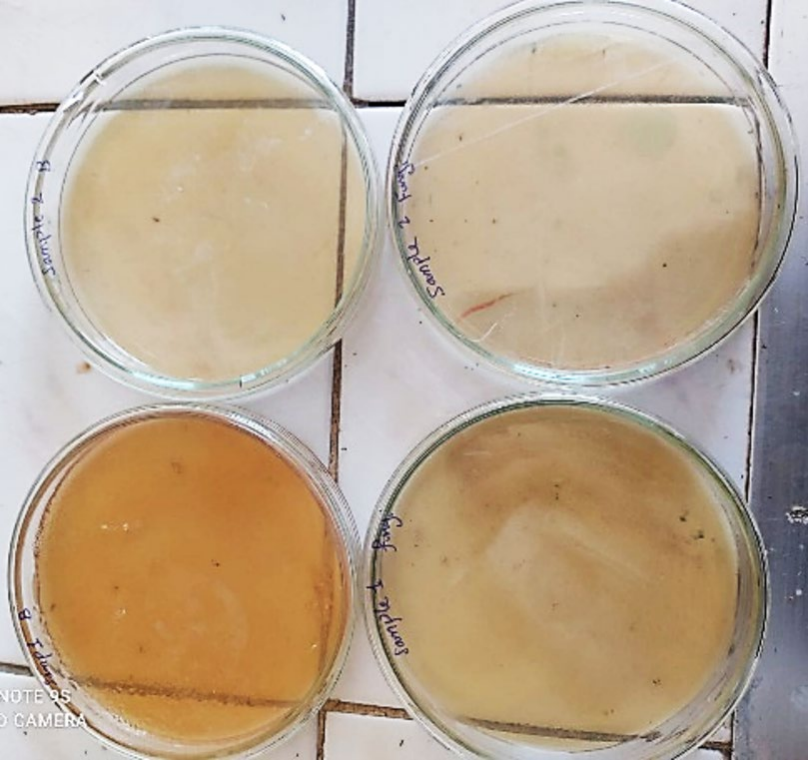
Showing effect of wastewater treated with Co@Ag, no growth for fungal or bacterial growth on different media used.

### Application of treated waste water

**Fig. 6:**
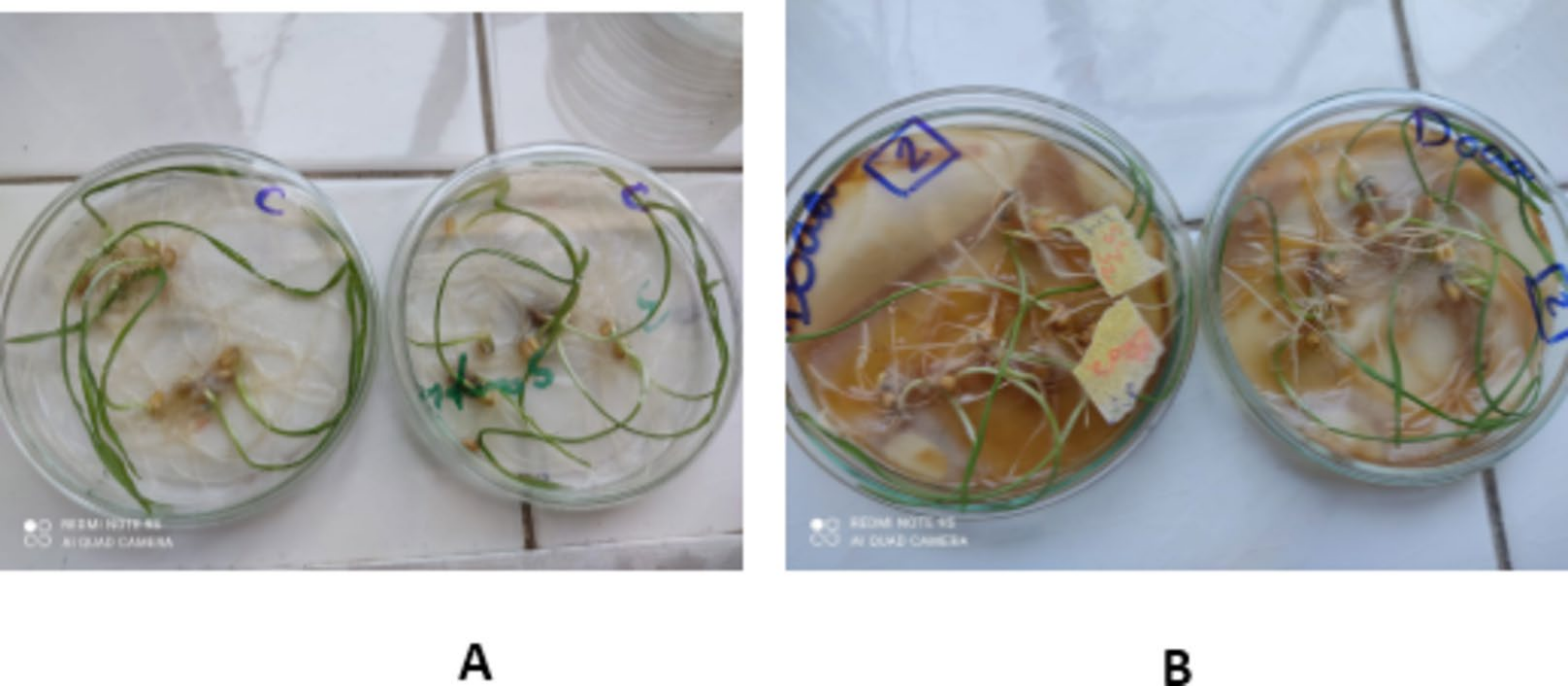
Effect of treated water samples by nanoparticles on wheat seeds growth, **A**: control (tap water), **B**: 20 mg/l concentration of Co@Ag on growth parameters.

Nanoparticles improve water quality, where it inhibit and have strong biocidal activity against bacterial and fungal pathogens growth, so treated wastewater become rich in nutrients and minerals needed by wheat plant growth without any microbial pathogen. The data presented in Table 4, **and** Fig. 6 showed the effects of water samples treated with varying Co@Ag concentrations on the shoot length, root length, and percent of germination of wheat seedlings. When plants irrigated with treated water samples with varying Co@Ag concentrations were applied foliarly, all these growth parameters increased in comparison to the plants irrigated with waste water. Data showed that when Co@Ag concentrations were raised from 0 to 10 and 20 mg/l, there was a progressive rise in shoot length, root length, and percent of germination of wheat seedlings. With 20 mg/l Co@Ag, the greatest response across all growth criteria was attained.

**Table 4:** Effect of waste water samples and treated samples with different concentrations of Co@Ag on growth of wheat seeds with statistical analysis.

Concentration of Co@Ag (mg/l)	Shoot length (cm) Water sample1 Water sample 2	Root length (cm) Water sample1 Water sample 2	Percent of germination Water sample1 Water sample 2
Tap water (control)	7.5^b^ ± 0.5 cm 7.5^b^ ± 0.5 cm	9^b^ ± 0.1 cm 9^b^ ± 0.1 cm	90% 90%
Waste water	2.4^a^ ± 0.5 cm 2.2^a^ ± 0.5 cm	2.9^a^ ± 0.5 cm 2.4^a^ ± 0.5 cm	30% 30%
10	8.1^b^ ± 0.1 cm 8.5^c^ ± 0.1 cm	9.6^c^ ± 0.1 cm 9.5^c^ ± 0.1 cm	100% 100%
20	9.2^c^ ± 0.1 cm 9.7^d^ ± 0.1 cm	11^d^ ± 0.1 cm 12.5^d^ ± 0.1 cm	100% 100%

The mean is for triplicate measurements from one independent experiment ± SD, a-d means with different superscripts in the same column are considered statistically different (Duncan’s multiple range test, p˂0.0001).

## Discussion

The selection of silver nanoparticles for this study was based on their high metal adsorption capacity, ease of synthesis, low cost of starting materials, and ability to purify water without producing sludge. The benefits of superparamagnetism and similar properties are shared by cobalt-silver nanoparticles, which also exhibit strong antibacterial activity, may be used to treat water, have an easy recovery process, cannot contaminate people with silver, and have a wide range of potential applications. Cobalt and silver particles act as heavy metals that have an oligodynamic and toxic activity against microbial growth, both cause complete damage of cell wall structure and ion leakage, silver attaching itself to important enzyme functional groups. The bacterial plasma or cytoplasmic membrane, which is linked to numerous vital enzymes, is a crucial target site for silver ions since they cause the release of K + ions from bacteria and cobalt, In the presence of hydrogen peroxide, cobalt damages DNA and produces reactive oxygen species. Synergistic effect of both ions occurs due to complementary mode of action of these ions on membrane, DNA and enzymes of microbial cells which in turn led to damage of microbial cells.

Our results showed that Co@Ag nanoparticles have antimicrobial activity against Gram negative and pathogenic fungi and this in agreement with^33^ where *B. Subtilis* and *Pseudomonas*Sp. infections were controlled by the Co NPs acting as a possible antiseptic. Also^34^, suggest that metal nanoparticles, both alone and in combination, have a strong antibacterial effect on both gram-positive and gram-negative bacteria. Cobalt, silver, and zinc combinations (Co + Zn, Co + Ag, Ag + Zn) shown a synergistic impact against both strains when coupled metal nanoparticles were used. By showing and counting the amount of separated bacterial and fungal isolates from wastewater treated with Co@Ag and demonstrating that there is no growth for bacteria after five days and for fungi after eight days, several types of selective and differential media are used. This results in accordance with a large portion of AgNP research is concerned with wastewater treatment, dye removal, and water purification. It focuses on the antibacterial action against many types of microbes^35^. In our results, that shoot and root length and germination percentage of wheat seeds irrigated by treated water increased progressively as Co@Ag concentrations were elevated from 0 to 10 and 20 mg/l, and this in agreement with^36^ who showed that in order to prevent leaf abscission, promote growth, and raise the survival rate of plantlets in the nursery stage, cobalt and silver nanoparticles (CoNPs, AgNPs) were employed, CoNPs aid in the reduction of ethylene gas concentration and the hydrolytic activity of enzymes including cellulase and pectinase. AgNPs was the best component for the bulk proliferation of shoots. And who showed that AgNPs) and cobalt nanoparticles (CoNPs) enhanced hoot multiplication rooting, acclimatization, growth and flowering of *Gerbera* (*Gerbera jamesonii*Revolution Yellow)^37^. The bulk material and the nanoparticles have quite different physicochemical characteristics. The nanoparticles react with environmental components to a large degree because of their increased reactivity. Nanoparticles affect the environment in two different ways. Although nanoparticles have the potential to cure environmental contaminants, they may also produce ecotoxicity. Nanoparticles’ effects on the environment are determined by the course and procedure of production and environmental stability of nanoparticles. It also relies on the nanoparticles’ physicochemical characteristics and capacity to accumulate in the environment^38^.The size, kind, and concentration of the nanoparticles as well as the kind of soil, plant species, hydraulic conductivity, and the availability of vital nutrients in the soil all affect the degree of toxicity and the effects of Co and AgNPs accumulation on the environment and plants^39,40^.

## Conclusion

The research emphasizes the possibility of using Cobalt silver nanoparticles (Co@Ag-NPs) as a sustainable and effective way to remediate wastewater, where it showed an antimicrobial activity against bacterial and fungal pathogens isolated from wastewater at different concentrations (10^−1^, 10^−2^, 10^−3^), after treatment of wastewater with 20 mg/l Co@Ag, the greatest response across all growth criteria in the tested plant was attained. Chemically synthesized Co@Ag-NPs are a low-toxicity, economical, and environmentally friendly wastewater treatment method. The wheat seeds benefited from the treated wastewater’s promotion of growth and germination rates. Fresh weight of roots and shoots increased with higher chlorophyll concentration. These results highlight the potential environmental benefits, sustainability, and cost-effectiveness of treating wastewater with Co@Ag NPs. By successfully lowering the levels of pollutants and phytotoxicity in the wastewater that has been treated, this method shows great promise in mitigating water pollution issues and boosting agricultural output. In order to ensure the preservation of ecosystems and public health, this research is crucial for assessing the environmental safety and sustainability of wastewater treatment techniques based on Co@Ag NPs. It also helps to guide decision-making and regulatory actions.

## Data Availability

The datasets used and/or analysed during the current study are available from the corresponding author on reasonable request.
